# Possibilities and limits for using the gut microbiome to improve captive animal health

**DOI:** 10.1186/s42523-021-00155-8

**Published:** 2021-12-29

**Authors:** Jessica Diaz, Aspen T. Reese

**Affiliations:** 1 Section of Ecology, Behavior, and Evolution, University of California, San Diego, 9500 Gilman Drive, La Jolla, CA 92093 USA; 2 Center for Microbiome Innovation, University of California, San Diego, 9500 Gilman Drive, La Jolla, CA 92093 USA

**Keywords:** Captivity, Gut microbiome, Animal, Wild, Conservation, Function

## Abstract

Because of its potential to modulate host health, the gut microbiome of captive animals has become an increasingly important area of research. In this paper, we review the current literature comparing the gut microbiomes of wild and captive animals, as well as experiments tracking the microbiome when animals are moved between wild and captive environments. As a whole, these studies report highly idiosyncratic results with significant differences in the effect of captivity on the gut microbiome between host species. While a few studies have analyzed the functional capacity of captive microbiomes, there has been little research directly addressing the health consequences of captive microbiomes. Therefore, the current body of literature cannot broadly answer what costs, if any, arise from having a captive microbiome in captivity. Addressing this outstanding question will be critical to determining whether it is worth pursuing microbial manipulations as a conservation tool. To stimulate the next wave of research which can tie the captive microbiome to functional and health impacts, we outline a wide range of tools that can be used to manipulate the microbiome in captivity and suggest a variety of methods for measuring the impact of such manipulation preceding therapeutic use. Altogether, we caution researchers against generalizing results between host species given the variability in gut community responses to captivity and highlight the need to understand what role the gut microbiome plays in captive animal health before putting microbiome manipulations broadly into practice.

## Introduction

In captivity, wild animals are subject to environments and lifestyles that they would not experience in their natural habitat as a result of significant human manipulation of the animals’ built environment, diet, healthcare, and social interactions [1]. These novel circumstances can lead to improved animal welfare and longevity in some species [2], but other species suffer from serious health problems under human care and human environments [2, 3]. There are diverse health problems which have been associated with captivity including issues with metabolism and digestion [4, 5], infection [6, 7], stress [8, 9], and reproduction [10, 11]. Reflecting its growing importance in other fields of organismal research, the microbiome has been proposed as a mediator of host condition under captivity [e.g. 12]. However, the extent, predictability, and drivers of microbiome changes under captivity remain unclear, limiting our ability to utilize microbial interventions to alleviate captivity-associated health problems.

Given the vast number of environmental variables that differ between captive and wild environments, it is difficult to predict how a particular species will fare in captivity [2]. The potential improvements (including veterinary care, freedom from predators, and increased availability of food [2]) and potential stressors (reduced range and mobility, artificial social groups, frequent proximity and contact with humans, etc. [3]) under captivity are numerous and hard to isolate from one another. Moreover, physical and behavioral differences between species may modulate the impact of certain changes and lead to varying health outcomes. For example, species that are typically solitary in the wild likely face different challenges than gregarious species when placed into captive environments with set social groups [13].

Microbial plasticity or heterogeneous microbial sensitivity could also contribute to differential animal responses under captivity. Diet and environment are known to shape the composition of the gut microbiome [e.g. 14, 15] with downstream effects on host metabolic, immune, and neurological systems in humans and animal hosts [16–18]. However, not all microbiomes respond similarly or to the same extent when exposed to new diets or housing conditions, and not all hosts will experience physiological effects as a result of microbiome plasticity [19, 20]. For example, domestication has been found to consistently impact the gut microbiota, but there is no singular domesticated gut microbiota [21]. Diet and captive habitats, as well as evolutionary change, can contribute to domestication related microbial shifts; for instance, wild horses kept in zoos show greater gut microbial similarity to domesticated horses than do wild animals in their native habitat [22].

Given the potential connection of gut microbial changes to downstream health effects and the high prevalence of gastrointestinal issues in captive animals (such as helicobacter gastritis seen in most captive cheetahs [23, 24], callitrichid wasting syndrome observed in various New World monkeys [4, 25], and *Cryptosporidium* infection that affects captive sifakas [26]), some have suggested including the microbiome in conservation management plans for captive animals [e.g. 27, 28]. These suggestions are inspired by strong effects of captivity observed in comparisons between wild and captive animal microbiomes. The generalizability of captivity effects on the microbiome and their connection to host health remain unclear, however, because to date there has not been a comprehensive review of the literature.

One of the particular challenges of studying health in response to the microbiome is that health is difficult to define and measure and varies on a species-by-species basis. Health extends beyond survival and reproduction and encompasses many axes of physical and mental well-being. In this paper we refer to health broadly, much like Fisher’s definition of animal welfare as “an evaluative term […] encompassing aspects of natural selection, coping, well-being, satisfaction of preferences, fulfilment of needs, and natural behaviour, or a combination of them” [29]. We emphasize that indicators of health and disease will vary between species, and there is no single definition of host health much like there is no single definition of a healthy microbiome [30]. For example, for a species struggling with gastrointestinal disease such as the red wolf [31], improved health may be defined as decreased instances of disease. Meanwhile, for a species that suffers from captive infertility such as the southern white rhinoceros [32], improved health may simply mean increased fecundity.

In this paper, we intend to help identify and address gaps in our understanding of the captive gut microbiome and in turn host health. We review the literature on how and why the gut microbiome responds to captivity, as well as what is known about the health consequences of these changes. To conclude, we highlight major outstanding questions in the field brought to light by our review and propose priority areas for future work, with an emphasis on research relevant to conservation efforts.

## Review of current literature on the captive microbiome

The scope of captivity’s influence on the gut microbiome can be summarized with three broad questions. How does the microbiome change in response to captivity? Why does it change? And, do these changes affect overall animal health for the better or for the worse? We focus here on the gut microbiome, which has been the subject of the preponderance of work on captive microbiomes, but note that an important exception considered elsewhere [e.g. 33, 34] is the amphibian skin microbiome.

### How does the gut microbiome change in response to captivity?

There have been more than 60 studies over recent decades characterizing the effects of captivity on the gut microbiome of host species spanning the animal kingdom. Though the term “captivity” can refer to a variety of habitats such as zoos, sanctuaries, labs, farms, or even households, most of these studies examine captivity in the context of zoos or sanctuaries and sometimes, but not always, focus on endangered species. Altogether, it is clear that there is no singular “captive microbiome,” as differences in microbial diversity and community composition between captive and wild environments vary greatly among hosts (Table 1).

**Table 1:** Papers comparing the gut microbiomes of captive animals and their wild counterparts

	Author	Year	Citation	Sample type	Species	Difference in captive/wild microbial diversity?	Difference in captive/wild microbial composition?	Functional implications discussed?
Mammals	Alfano et al.	2015	[113]	rectal	Koala	Not reported	N	Not discussed
	Allan et al.	2018	[114]	fecal	Amargosa vole	No difference	Y	Discussed
	Allan et al.	2018	[114]	foregut	Amargosa vole	No difference	N	Discussed (metabolic function)
	Amato et al.	2016	[5]	fecal and intestinal	Various colobine species	No difference	Y	Discuss (metabolic function)
	Benno et al.	1987	[115]	fecal	Japanese macaque	Higher in captivity	Y	Discussed (metabolic function)
	Bik et al.	2016	[116]	rectal	Bottlenose dolphin	Not reported	N	Not discussed
	Borbon-Garcia et al.	2017	[73]	fecal	Andean bear	Lower in captivity	Y	Assessed (predicted metabolic functions—KEGG pathways/PICRUSt)
	Cheng et al.	2015	[72]	fecal	Tasmanian devil	Lower in captivity	Y	Assessed (predicted metabolic functions—KEGG pathways/PICRUSt)
	Clayton et al.	2018	[44]	fecal	Red-shanked douc	Lower in captivity	Y	Assessed (predicted metabolic and antibiotic resistance functions—KEGG pathways/PICRUSt)
	Clayton et al.	2016	[55]	fecal	Red-shanked douc, Mantled howling monkey	Lower in captivity	Y	Assessed (predicted metabolic functions—PICRUSt)
	De Jesus-Laboy et al.	2011	[117]	fecal	Goat	Not reported	N	Assessed (Assess presence of antibiotic resistance genes)
	Delport et al.	2016	[118]	fecal	Australian sea lion	Not reported	Y	Not discussed
	Delsuc et al.	2013	[119]	fecal	Various myrmecophagous mammals	Not reported	Y	Not discussed
	Eigeland et al.	2012	[120]	fecal	Dugong	Lower in captivity	Y	Not discussed
	Eisenhofer et al.	2021	[35]	fecal	Southern hairy-nosed wombat	Lower in captivity	Y	Discussed (metabolic function)
	Frankel et al.	2019	[121]	fecal	5 primate species	Lower in captivity	Y	Discussed (metabolic function)
	Gao et al.	2019	[87]	fecal	Tibetan wild ass	Lower in captivity	Y	Discussed (immune function)
	Gibson et al.	2019	[38]	fecal	Black rhinoceros	No difference	Y	Assessed (functional metagenomics—metabolic)
	Greene et al.	2019	[20]	fecal	Various lemur species	Not reported	Y	Discussed (metabolic function)
	Guan et al.	2016	[122]	fecal	Sable	Not reported	Y	Discussed (metabolic function)
	Guan et al.	2017	[123]	fecal	Sika deer	Higher in captivity	Y	Discussed (metabolic function)
	Guo et al.	2019	[124]	fecal	Giant Panda	Lower in captivity	Y	Assessed (functional metagenomics—metabolic and immune)
	Hale et al.	2019	[125]	fecal	Snub-nosed monkey	Lower in captivity	Y	Discussed (metabolic and immune function)
	Haworth et al.	2019	[126]	fecal	Mountain goat	No difference	Y	Discussed
	Kong et al.	2014	[127]	fecal	Red panda	Lower in captivity	Y	Assessed (associated present OTUs with cellulose degradation ability (GenBank/Kimura—phylogenetic analysis based on 16S))
	Li et al.	2017	[86]	fecal	Forest musk deer	No difference	Y	Discussed (metabolic function)
	McKenzie et al.	2017	[42]	fecal	41 mammal species	Inconsistent between species	Y (except even-toed ungulates)	Discussed (metabolic function)
	Metcalf et al.	2017	[22]	fecal	Przewalski's horse	Lower in captivity	Y	Not discussed
	Milovic et al.	2020	[128]	fecal	White-footed mouse	Lower in captivity	Y	Not discussed
	Minich et al.	2021	[78]	fecal	White-tailed deer	Higher in captivity	Y	Discussed (metabolic and immune function)
	Moustafa et al.	2021	[129]	fecal	Asian elephant	No difference	Y	Discussed (metabolic function)
	Nakamura et al.	2011	[130]	fecal	Black howler monkey	Lower in captivity	Y	Discussed (metabolic function)
	Narat et al.	2020	[58]	fecal	Chimpanzee	No difference	Y	Discussed (metabolic function)
	Narat et al.	2020	[58]	fecal	Western lowland gorilla	Higher in captivity	Y	Discussed (metabolic function)
	Nelson et al.	2012	[39]	fecal	Elephant seal and Leopard seal	Higher in captivity	Y	Discussed (immune function)
	Ning et al.	2020	[88]	fecal	Amur Tiger	Higher in captivity	Y	Assessed (functional metagenomics—metabolic)
	Prabhu et al.	2020	[131]	fecal	Gaur	No difference	Y	Assessed (predicted metabolic and immune functions—PICRUSt/KEGG)
	Rosshart et al.	2017	[79]	ileocecal	House mouse	Not reported	Y	Assessed (transplant experiment with immune readouts)
	Schwab et al.	2011	[132]	fecal	Grizzly bear	Not reported	Y	Discussed (immune function)
	Sun et al.	2019	[74]	fecal	Alpine musk deer	Not reported	Y	Assessed (functional metagenomics—metabolic)
	Sun et al.	2019	[76]	fecal	Père David’s deer	No difference	Y	Assessed (predicted metabolic functions—PICRUSt/KEGG)
	Tang et al.	2020	[133]	fecal	Giant panda	Lower in captivity	Y	Discussed (metabolic and immune function)
	Tsukayama et al.	2018	[40]	fecal	Kinda and grayfoot chacma baboon	Higher in captivity	Y	Assessed (functional metagenomics—abx resistance)
	Uenishi et al.	2007	[134]	fecal	Chimpanzee	Not reported	Y	Discussed (metabolic function)
	Wasimuddin et al.	2017	[135]	fecal	Cheetah	No difference	Y	Assessed (predicted metabolic and immune functions—PICRUSt/KEGG)
	Xiao et al.	2019	[136]	fecal	6 bat species	Higher in captivity	Did not compare	Assessed (predicted metabolic functions—PICRUSt/KEGG)
	Yan et al.	2021	[9]	fecal	Pangolin	Higher in captivity	Y	Not discussed
Birds	Oliveira et al.	2020	[37]	fecal	Various raptor species	No difference	Y	Not discussed
	San Juan et al.	2021	[137]	fecal	Brown kiwi	Lower in captivity	Y	Not discussed
	Scupham et al.	2008	[138]	cecal	Turkey	No difference	Y	Discussed (metabolic function)
	Ushida et al.	2016	[6]	cecal	Japanese and Svalbard rock ptarmigan	Not reported	Y	Discussed (metabolic function)
	Wienemann et al.	2011	[36]	cecal	Capercaillie	Lower in captivity	Y	Discussed (metabolic function)
	Xenoulis et al.	2010	[41]	cloacal	3 parrot species	Higher in captivity	Y	Not discussed
	Xie et al.	2016	[139]	fecal	Red-crowned crane	Higher in captivity	Y	Discussed (immune function)
Reptiles	Campos et al.	2018	[140]	fecal and rectal	Green turtle	No difference	N	Discussed (metabolic function)
	Garcia-De la Pena et al.	2019	[141]	fecal	Bolson tortoise	No difference	Y	Discussed (metabolic function)
	Sandri et al.	2020	[142]	fecal	Aldabra giant tortoise	No difference	Y	Discussed (metabolic function)
	Tang et al.	2020	[143]	fecal	Crocodile lizard	Higher in captivity	Y	Assessed (predicted metabolic functions—PICRUSt/KEGG)
Amphibians	Tong et al.	2019	[144]	intestinal	Dybowski's brown frog	No difference	Y	Assessed (predicted metabolic and immune functions—PICRUSt/KEGG)

Included is whether the paper reported a difference in microbial diversity and microbial composition, as well as whether functional implications of these differences were discussed or assessed. Papers were found with a directed review of existing literature including a Google Scholar search and consulting references cited in each paper collected. We retained only those studies which include gut microbiome measurements of at least one population of captive and one population of wild vertebrates

Many studies that measured microbial diversity report decreased gut richness or diversity in individuals in captivity compared to others in the wild [e.g. 22, 35, 36]. However, some studies have reported no difference in microbial diversity between the two environments [e.g. 37, 38], or even increased diversity in captive animals [e.g. 39–41]. The most comprehensive test of captivity effects to date demonstrates this heterogeneity persists even when sampling and sequencing methods are controlled for [42]. Of the 11 mammalian families represented in that analysis, only four showed significantly decreased bacterial diversity in captivity. Six other families showed no significant difference between the captive and wild states, and one showed significantly increased diversity in captivity. A recent meta-analysis of 23 comparative studies lends further support to this ambiguity, documenting no systematic effects of captivity on gut microbial diversity [43].

Similar heterogeneity has been observed in comparative analyses of microbial community composition. With few exceptions, comparative studies typically show that overall microbial composition differs significantly between captive and wild populations (Table 1). Yet, there is no apparent consistency across host species as to whether certain bacterial taxa are enriched or depleted in captivity. While it is difficult to compare papers in which only a single host species was sampled because of differences in study design and data analysis, there are a few studies that have looked at microbial composition in several captive species at once. In primates, two studies have documented a convergence toward human-like microbiomes in captivity [44, 45]. The 41-species comparison by McKenzie et al. documented no convergence and noted variable shifts in the relative abundance of bacterial phyla between host genera [42]. Finally, in a study involving multiple lemur species, Greene et al. observed cases where one or the other of the prominent bacterial genera *Bacteroides* and *Prevotella* increased in captivity depending on the dietary strategy of the host [20].

Beyond comparative work, there is limited data tracking the trajectory of microbial change in individual animals moved into captivity from the wild. Here too, though, variable microbial responses to captivity have been observed between host species (Table 2). Findings are inconsistent as to whether gut microbial diversity increases or decreases when animals are brought into captivity as well as how community composition changes. For example, Kohl et al. transplanted two closely related species of woodrats from the wild to captivity and noted striking differences between the species in the magnitude that microbial diversity decreased, as well as opposing trends in the relative abundance of bacteria in the phyla Proteobacteria and Firmicutes [19]. The gut microbiome can respond quickly upon introduction to captivity—for example Kohl and Dearing documented compositional changes in captive woodrats beginning within two weeks of capture [46]—but in some cases wild microbiome characteristics last much longer. A large proportion of the wild microbiome in rodents and lizards is retained in captivity through at least several months [19, 46, 47], and wild mouse microbial signatures can be transmitted through at least 10 generations of captive breeding in a laboratory facility [48].

**Table 2:** Papers comparing gut microbiota before and after experimental transplantation from the wild to captivity

	Author	Year	Citation	Sample type	Species	Difference in captive/wild microbial diversity?	Difference in captive/wild microbial composition?	Functional implications discussed?
Mammals	Edenborough et al.	2020	[145]	Fecal	Angolan free-tailed bat	Higher in captivity	Y	Not discussed
	Kohl and Dearing	2014	[46]	Fecal	Desert woodrat	No difference	N	Not discussed
	Kohl et al.	2014	[19]	Fecal	White-throated and Stephen's woodrat	Lower in captivity	Y	Assessed (metabolic function—monitored ability to digest natural diet)
	Schmidt et al.	2019	[146]	Fecal	Deer mouse	Lower in captivity	Y	Discussed (metabolic function)
Other	Dhanasiri et al.	2010	[147]	mid and posterior large intestine	Atlantic cod	No difference	Y	Discussed (immune function)
	Kohl et al.	2017	[47]	Fecal	3 lizard species	No difference	Y	Discussed (metabolic function)

Included is whether the paper reported an increase or decrease in microbial diversity and microbial composition, as well as whether functional implications of these differences were discussed or assessed. Papers were found with a directed review of existing literature including a Google Scholar search and consulting references cited in each paper collected. We retained only those studies which include a wild population brought into captivity within a single lifetime

A few researchers have conducted the inverse transplant experiment, tracking the microbiome as captive animals are reintroduced to the wild (Table 3). These studies provide some evidence that the captive gut microbiome shifts back to resemble the wild gut microbiome after reintroduction and can do so on the timescale of days to weeks [e.g. 49–51]. But, the observed shift back towards a wild microbiome may not completely erase microbial signatures of an animal’s captive origin, as seen in Przewalski’s horses even after 10 years post-release [22]. It is worth noting compositional shifts following reintroduction are likely highly dependent on the environment to which animals are transplanted. In addition to moving animals from captivity to the wild, Chong et al. transplanted Tasmanian Devils from one wild location to another and saw a compositional shift towards a microbiome that resembled native Tasmanian Devils of the new location [50]. The lack of a universal “wild” microbiome, even for a single species, is ubiquitous and logical given findings that some gut bacteria may be geographically restricted or fluctuate seasonally [30, 52–54].

**Table 3:** Papers comparing gut microbiota before and after experimental transplantation from captivity to the wild

	Author	Year	Citation	Sample type	Species	Difference in captive/wild microbial diversity?	Difference in captive/wild microbial composition?	Functional implications discussed?
Mammals	Bar et al.	2020	[49]	Fecal	House mouse	Lower in captivity	Y	Discussed (immune function)
	Chong et al.	2019	[50]	Fecal	Tasmanian devil	Lower in captivity	Y	Discussed (metabolic function)
	Leeuwen et al.	2020	[51]	Fecal	Deer mouse	No difference	Y	Discussed (neurological function)
	Schmidt et al.	2019	[146]	Fecal	Deer mouse	Lower in captivity	Y	Discussed (metabolic function)
	Yao et al.	2019	[75]	Fecal	Giant panda	Lower in captivity	Y	Assessed (functional metagenomics)

Included is whether the paper reported an increase or decrease in microbial diversity and microbial composition, as well as whether functional implications of these differences were discussed or assessed. Papers were found with a directed review of existing literature including a Google Scholar search and consulting references cited in each paper collected. We retained only those studies which include a captive population released into the wild within a single lifetime

### What drives gut microbiome changes in captivity?

While it is clear that transitioning between wild and captive conditions can elicit changes to the gut microbiome, the ecological factors altered under captivity that specifically cause microbiome shifts often remain unspecified. Captive diets can vary widely from natural diets [3, 55], and diet has been shown to have a strong influence over gut microbial composition in captive animals [21, 31, 51, 56]. Even minor changes in diet can lead to shifts in microbial composition, as evidenced in captive sifakas [57]. Environmental conditions beyond diet can also matter, with one study demonstrating that rehousing lab mice in a room with altered temperature and humidity, but the same diet, resulted in significant changes in gut microbial composition [49]. Additionally, microbial dispersal from abiotic environmental substrates (water, soil, plants, etc.), as well as from contact with conspecifics or other species (including humans), can influence the gut microbiome of captive animals and can vary between captive facilities [58]. Komodo dragons, for example, harbor gut microbes that are also present in their enclosures [59], while human handling has been implicated in the spread of microbes between mouse enclosures [48]. Animal social groups have a strong influence on gut microbial composition in the wild, likely affecting the microbiome by transferring bacteria between individuals through physical contact among social networks [e.g. 60–62]. It is reasonable to expect similar social dynamics transmit microbes among captive populations, although, beyond studies of cohousing [e.g. 63, 64], this is rarely explicitly considered.

It must be noted that diet and environmental exposures can be tightly linked, especially in wild environments, making it challenging to disentangle their microbial impacts. Diet has the potential to have both a probiotic and prebiotic influence on the microbiome as it exposes animals to environmental microbes in addition to providing direct substrates for bacterial growth [52, 65]. Animal diets also often vary as environmental conditions change. One study, for example, saw a strong effect of the amount of outdoor exposure on changes to the gut microbiome of colobine primates, but it could not be determined whether this was a result of the animals being exposed to new environmental microbes or eating a greater diversity of foliage [55].

Interaction with humans introduces additional stressors unique to the captive environment that can influence the microbiome. Most notably, administration of antibiotics reduces the natural flora of the gut and may even compound the effects of captivity by quickly removing large portions of the wild microbiota [66]. Isolation from natural pathogens like helminths through anthelminthic medicine or increased hygiene can induce changes to the microbiome by affecting the immune system [67, 68]. Additionally, direct physical interactions with humans, such as keepers or visitors, could both directly introduce human-associated microbes [44, 45] and cause stress, which itself can induce shifts in microbial composition [e.g. 69, 70].

### Do gut microbial compositional changes in captivity affect microbiome function or host health? If so, how?

While the reviewed studies clearly show that captivity can affect the composition and diversity of the gut microbiome, characterizing these two metrics is insufficient to determine whether the gut microbiome plays a role in captive animal health as they do not give insight into whether bacterial functions change [71]. To date, there have been few studies that compare functional capacity of the microbiome in the wild and in captivity in addition to composition. Of these, most have predicted functionality using reference genomes related to taxa represented in 16S rRNA gene sequencing data [e.g. 44, 72, 73] and a few have conducted functional metagenomic sequencing [e.g. 38, 40, 74, 75]. Most show that predicted function differs between captive and wild microbiomes (but see [76] for a case in which no functional difference was predicted) and argue the altered functional capacity of the bacterial community may have a realized impact on host biology. However, altered functional capacity does not necessarily indicate changes in bacterial activity or host physiology [77].

There have been some instances, though, where microbial composition has been more directly tied to health outcome in captive animals. A handful of studies have correlated gut microbiome composition to specific disease phenotypes in captive animals, including chronic wasting disease in deer [78], gastrointestinal disease in wolves [31], and iron overload disorder in rhinoceroses [7]. That work provides support for a functional connection between microbial composition and health consequences in at least some contexts. Bolstering the idea that the wild microbiome specifically can be beneficial, fecal microbiota transplantation (FMT) of a wild microbiota community has been used in mammals to successfully reduce inflammation and improve survival against viral disease [79] and expand dietary niche [80], and probiotic supplementation improved body weight and fecal quality in cheetahs [81]. In most cases, however, the potential for microbial manipulations to lead to favorable health outcomes have not been explicitly tested, so the generalizability of these findings remains unknown. Microbial manipulations in captive livestock have also been used to try to address health concerns, although the mixed success of FMTs and dietary interventions in agricultural contexts underscores the likelihood that an altered microbiota will not cure all ills [82–85].

There are alternative interpretations of microbial differences between wild and captive animals that do not presume the captive microbiome is less favorable [e.g. 35, 86–88] or potentially detrimental [1]; these are important to consider in the absence of species-specific experimental evidence. First, microbiomes may have changed to match the new captive environment in a beneficial manner [89]. For example, natural selection could promote a microbiome better able to digest the captive diet while potentially reducing the animal’s capacity to digest a (no longer relevant) wild diet [21, 90]. Captive microbiome contributions to promoting growth or mitigating stressors may not fully outweigh the costs of a captive lifestyle, so captive animals may still be found to have worse health than wild individuals. Still, in these cases a return of a wild or ancestral microbiome to a captive animal would further diminish health in captivity. Second, microbiome changes under captivity may be constrained by tradeoffs such that the increase in some microbial functions, such as those relevant to captive environments, lead to the decrease or loss of other functions [77]. If so, re-establishing a wild microbiome may improve some aspects of health while reducing others. Third, the captive microbiome may be caused by, rather than causative of, poor health, in which case treating the microbiome would not alter the underlying condition. Finally, changes in the microbiome under captivity may primarily be neutral given functional redundancy of gut microbiota [91]. Given the scope of the current evidence that exists, we cannot presume to know what effect the captive microbiome has on the health of any given species. To ensure our understanding is sufficient for conservation applications, more research must be done to indicate under which circumstances and for which species microbial manipulations can improve health.

## Future directions

There is no single answer to the question of whether the gut microbiome matters for host health in captivity in large part because there is no single captive microbiome. The microbiome’s impact will be particular to host species, diet, external environment, conservation status, and reintroduction plans, among many other factors that impact the gut microbiome and/or affect species health priorities. Insofar as there is a prospect of health-relevant compositional and functional differences of the gut microbiome under captivity, we must identify when reconstituting a wild microbiome is most likely to be a suitable route for improving animal welfare and conservation. (Although, even absent demonstrated health benefits, there may be cases where reconstituting a wild microbiome is a worthwhile goal, for instance where it provides more ecologically realistic models for host-microbe interaction research.)

Responsible applications of gut microbiome interventions to animal conservation practices will depend on knowing whether the captive microbiome is truly involved in modulating a given health concern. The gold standard causal evidence requires experimental microbiome manipulations in captive animals: changing the microbiome either through fecal transplants, antibiotics, or probiotics then measuring host phenotypes of interest to assess whether existing health problems are ameliorated [28, 79–81, 92]. Similar experimental tests could be employed to assess whether microbial manipulations prior to reintroduction improve survival in the natural environment. Of course, experiments for observing direct causality have many potential ethical and logistical limitations, particularly if concerning endangered species, so may not be possible in many cases.

**Fig. 1:**
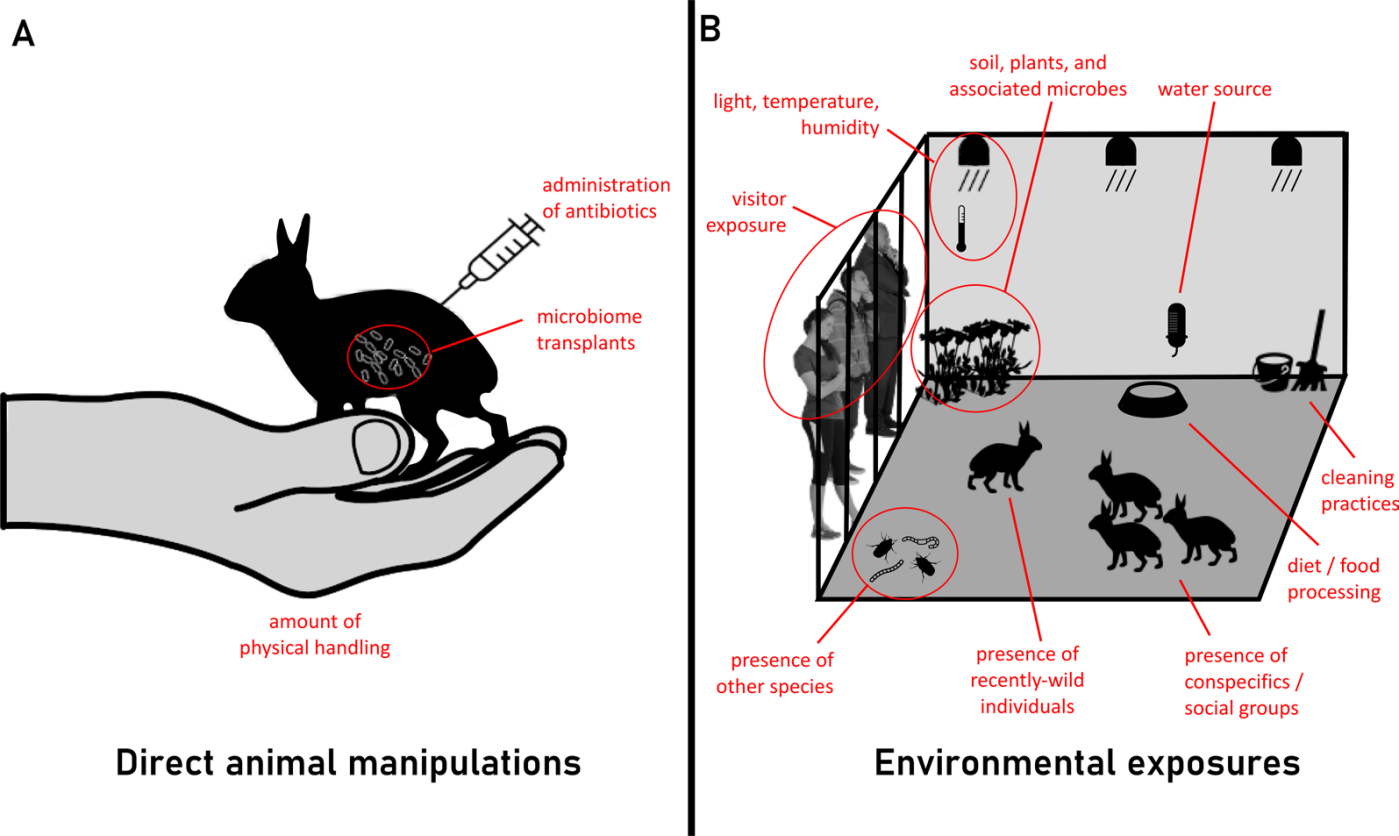
Possible interventions for manipulating the captive animal microbiome. **A** Direct animal manipulations include administration of antibiotics, microbiome transplants, and physical handling. **B** Manipulations targeting environmental exposures include presence of other animals (other species, conspecifics, and recently-wild individuals), diet and food processing, water source, cleaning practices, visitor exposure, climate, and soil, plants, and their associated microbes. Icons have been adapted under a Creative Commons license (https://creativecommons.org/licenses/by/3.0/) at phylopic.org and thenounproject.com. The hand image was sourced from clipart-library.com under a Personal Use license

Prior to proposing microbiome manipulations (either for experimental research or treatment purposes), alternate studies which go beyond documenting microbiome differences but not as far as microbial treatment could provide better corroboration of captivity-mediated microbiome health effects. Altering captive conditions to make the microbiome more wild-like (*e.g.*, restoring diet complexity, increasing exposure to other individuals or species, diversifying environmental substrates, limiting handling; see Fig. 1) then tracking microbial and host health responses could identify beneficial wild-associated taxa or other critical ecological drivers of the captive microbiome.

If experiments in the species of interest are not possible, alternatives include gnotobiotic or in vitro research. In gnotobiotic studies, gut microbiota from different donors, such as wild and captive individuals, are transplanted into germ-free animals (often mice) whose phenotypes are then compared [93, 94]. These experiments have their own limitations including expense and limited facilities, as well as the reduced biological realism that comes from measuring a microbiome’s effect in a mismatched host [95], but they could be particularly useful for studying the microbiome of endangered species. Similarly, in vitro culturing experiments could provide insight into microbial functioning of wild and captive gut microbiomes when live animal work is infeasible. Given that in vitro studies remove host biology, they cannot be used to relate gut microbial composition to host phenotype. However, they could allow researchers to characterize whole microbial communities under varying stimuli and measure functional responses relevant to host biology in a relatively cheap and more high-throughput manner [96, 97]. They may prove especially useful for testing potential manipulations (*e.g.*, diet, microbial exposure) for promoting a shift from a captive to more wild-like microbiota.

More comprehensive observational studies that tie microbiome composition to host phenotype data in wild and captive settings could prioritize host species or health conditions which warrant further study [e.g. 7, 9, 31, 78]. Correlations between health status and the microbiome would not distinguish between cases where the captive microbiome was responsible for altered health and ones where it is a response to the unhealthy state or is curbing potentially worse states. They would, however, advance the field beyond characterizing the microbiome in wild and captive states and serve for hypothesis-generation to motivate future experimental work. Correlative studies have the benefit of being less invasive and lower cost than direct manipulations but do require a robust sample set. In addition to collecting new data, researchers could leverage banked fecal samples and existing zoo or sanctuary records or larger databases like the Zoological Information Management System (ZIMS), Animal Records Keeping System (ARKS), Single Population Animal Records Keeping System (SPARKS), and Medical Animal Records Keeping System (MedARKS) [98].

Regardless of the approach chosen for a future study, choosing appropriate phenotypic markers to assess host health will be critical. Previous research on microbiome mediated health impacts has primarily been conducted in animal models or humans [e.g. 99–101], so common markers and their benchmarks may not be entirely applicable to wild species or wild contexts (but see [102, 103] for biomarkers that may be applicable to wild animals). While transcriptomic or metabolomic data are frequently collected in biomedical research and can illuminate molecular mechanism [77], data at that scale is often unnecessary for conservation work. Given the high cost and technical demands of “omics” measurements, alternatives such as stool consistency, parasitology, body size, or physical condition may be more appropriate. Indeed, some of these have been previously used to measure health phenotypes in wild or captive species [e.g. 31, 104–106], although not always with explicit ties to the microbiome. Pathological biomarkers targeted towards a particular health concern, for instance diagnosing or determining the severity of gastrointestinal issues, infection status, or neurological and stress disorders [e.g. 9, 107, 108], could be more informative but potentially more invasive or costly. Ultimately, markers should be chosen based on the species and health concern of focus, as well as the laboratory capabilities available. Furthermore, any research program studying the captive gut microbiome should be diligent about considering multiple axes of animal health when possible given the possibility for functional tradeoffs resulting from microbial compositional shifts [77].

Deciding how to implement these research strategies aimed at causation over correlation in studies of the captive gut microbiota will be highly dependent on logistical and scientific factors specific to a given research program. Limited resources, including access to animals, may come up against conservation priorities and severely restrict researchers’ choices. Fortunately, the methods presented above offer a wide range of options that may allow scientists to do functionally relevant research on the captive animal microbiome even when economic or logistical limitations preclude direct microbial manipulation experiments. Experts on specific animals will be best suited to decide which research strategies and phenotypic readouts will be most useful and feasible for achieving pressing conservation goals for the target species, as it is clear now that results will not be generalizable between taxa.

Once health links to the gut microbiome are identified for a given species, there are additional challenges in deciding whether and how to incorporate microbial manipulations into husbandry practices as this would require investments of both funding and labor at animal facilities. Again, species experts will be in the best position to balance wellness and conservation goals with the costs and challenges of microbial manipulations—particularly if functional tradeoffs are present. For example, if the captive microbiome proves beneficial in captivity but harmful when animals are reintroduced, the short-term wellbeing of an individual animal must be weighed against the long-term wellbeing of the species. To successfully reintroduce animals to the wild may require pre-seeding a wild microbiome, or pre-seeding may be an unnecessary expense if functionality recovers when the microbiome reverts to a more wild-like state on its own [22, 49–51]. It is likely that certain manipulations will be more appropriate or more costly for some species than others, and institutions such as zoos that manage a wide variety of species will likely need to employ a wide range of strategies to cope with this variation. Despite these challenges, the potential benefits of harnessing the influence of the gut microbiome to improve host health is undeniable and will certainly be a worthwhile research avenue for many species.

## Conclusion

As more species are becoming impacted by threats such as climate change, human land use, and emerging infectious diseases [109–112], finding new ways to improve animal welfare in captivity may be critical for species success through captive breeding and reintroduction programs. Much work has been done in this century to characterize the differences between gut microbial composition of captive animals and their wild counterparts. As a whole, it is clear that there is enormous variation in how species’ gut microbes respond to the captive environment. Thus, there will likely be variation in how the captive microbiome influences host health, preventing a one size fits all approach to managing animal health through the microbiome. Both captive microbiome research and eventual mitigation strategies will likely need to be handled on a species-by-species basis managed by those who are most familiar with their particular biology, but in all cases they should include broader surveys of health phenotypes that consider immune, behavioral, and reproductive health in addition to metabolic health.

This complexity raises important questions for how the field should move forward, such as what species should be prioritized for research and what aspects of host health should be optimized. For example, should we begin with species that are known to have poor health in captivity or with those that face the greatest extinction threats? And is it worth improving metabolic health in captivity if it comes at the cost of immune health or metabolic functioning in the wild? The answers to these questions will likely be influenced by logistical barriers such as species availability and population sizes, but they merit careful consideration by scientists continuing captive microbiome research. As the field moves away from characterizing microbial composition and focuses more on linking microbial function to host phenotypes, it is crucial that researchers not only consider these questions, but acknowledge the limitations of their findings to avoid recommending widespread microbial manipulations before their effects are fully understood.

## Data Availability

Not applicable.

## References

[1] Carthey AJR, Blumstein DT, Gallagher RV, Tetu SG, Gillings MR (2020). Conserving the holobiont. Funct Ecol.

[2] Mason GJ (2010). Species differences in responses to captivity: stress, welfare and the comparative method. Trends Ecol Evol.

[3] Morgan KN, Tromborg CT (2007). Sources of stress in captivity. Appl Anim Behav Sci.

[4] Gore MA, Brandes F, Kaup F-J, Lenzner R, Mothes T, Osman AA (2001). Callitrichid nutrition and food sensitivity. J Med Primatol.

[5] Amato KR, Metcalf JL, Song SJ, Hale VL, Clayton J, Ackermann G (2016). Using the gut microbiota as a novel tool for examining colobine primate GI health. Glob Ecol Conserv.

[6] Ushida K, Segawa T, Tsuchida S, Murata K (2016). Cecal bacterial communities in wild Japanese rock ptarmigans and captive Svalbard rock ptarmigans. J Vet Med Sci.

[7] Roth TL, Switzer A, Watanabe-Chailland M, Bik EM, Relman DA, Romick-Rosendale LE (2019). Reduced gut microbiome diversity and metabolome differences in rhinoceros species at risk for iron overload disorder. Front Microbiol.

[8] Seibert LM, Luescher AU (2006). Feather-picking disorder in pet birds. Manual of parrot behavior.

[9] Yan D, Hu D, Li K, Li B, Zeng X, Chen J (2021). Effects of chronic stress on the fecal microbiome of malayan pangolins (*Manis javanica*) rescued from the illegal wildlife trade. Curr Microbiol.

[10] Tubbs CW, Moley LA, Ivy JA, Metrione LC, LaClaire S, Felton RG (2016). Estrogenicity of captive southern white rhinoceros diets and their association with fertility. Gen Comp Endocrinol.

[11] Petter JJ, Martin RD (1975). Breeding of malagasy lemurs in captivity. Breeding endangered species in captivity.

[12] Bahrndorff S, Alemu T, Alemneh T, Lund NJ (2016). The microbiome of animals: implications for conservation biology. Int J Genomics.

[13] Price EE, Stoinski TS (2007). Group size: determinants in the wild and implications for the captive housing of wild mammals in zoos. Appl Anim Behav Sci.

[14] Carmody RN, Gerber GK, Luevano JM, Gatti DM, Somes L, Svenson KL (2015). Diet dominates host genotype in shaping the murine gut microbiota. Cell Host Microbe.

[15] Scepanovic P, Hodel F, Mondot S, Partula V, Byrd A, Hammer C (2019). A comprehensive assessment of demographic, environmental, and host genetic associations with gut microbiome diversity in healthy individuals. Microbiome.

[16] Diaz Heijtz R, Wang S, Anuar F, Qian Y, Björkholm B, Samuelsson A (2011). Normal gut microbiota modulates brain development and behavior. Proc Natl Acad Sci USA.

[17] Round JL, Mazmanian SK (2009). The gut microbiota shapes intestinal immune responses during health and disease. Nat Rev Immunol.

[18] Tremaroli V, Bäckhed F (2012). Functional interactions between the gut microbiota and host metabolism. Nature.

[19] Kohl KD, Skopec MM, Dearing MD (2014). Captivity results in disparate loss of gut microbial diversity in closely related hosts. Conserv Physiol.

[20] Greene LK, Bornbusch SL, McKenney EA, Harris RL, Gorvetzian SR, Yoder AD (2019). The importance of scale in comparative microbiome research: new insights from the gut and glands of captive and wild lemurs. Am J Primatol.

[21] Reese AT, Chadaideh KS, Diggins CE, Schell LD, Beckel M, Callahan P (2021). Effects of domestication on the gut microbiota parallel those of human industrialization. Elife.

[22] Metcalf JL, Song SJ, Morton JT, Weiss S, Seguin-Orlando A, Joly F (2017). Evaluating the impact of domestication and captivity on the horse gut microbiome. Sci Rep.

[23] Munson L (1993). Diseases of captive cheetahs (*Acinonyx jubatus*): results of the cheetah research council pathology survey, 1989–1992. Zoo Biol.

[24] Terio KA, Munson L, Moore PF (2012). Characterization of the gastric immune response in cheetahs (*Acinonyx jubatus*) with helicobacter-associated gastritis. Vet Pathol.

[25] Cabana F, Maguire R, Hsu C-D, Plowman A (2018). Identification of possible nutritional and stress risk factors in the development of marmoset wasting syndrome. Zoo Biol.

[26] McKenney EA, Greene LK, Drea CM, Yoder AD (2017). Down for the count: cryptosporidium infection depletes the gut microbiome in Coquerel’s sifakas. Microb Ecol Health Dis.

[27] West AG, Waite DW, Deines P, Bourne DG, Digby A, McKenzie VJ (2019). The microbiome in threatened species conservation. Biol Conserv.

[28] Trevelline BK, Fontaine SS, Hartup BK, Kohl KD (2019). Conservation biology needs a microbial renaissance: a call for the consideration of host-associated microbiota in wildlife management practices. Proc R Soc B Biol Sci.

[29] Fisher M (2009). Defining animal welfare—does consistency matter?. N Z Vet J.

[30] Eisenstein M (2020). The hunt for a healthy microbiome. Nature.

[31] Bragg M, Freeman EW, Lim HC, Songsasen N, Muletz-Wolz CR (2020). Gut microbiomes differ among dietary types and stool consistency in the captive red wolf (*Canis rufus*). Front Microbiol.

[32] Williams CL, Ybarra AR, Meredith AN, Durrant BS, Tubbs CW (2019). Gut microbiota and phytoestrogen-associated infertility in southern white rhinoceros. MBio.

[33] Becker MH, Richards-Zawacki CL, Gratwicke B, Belden LK (2014). The effect of captivity on the cutaneous bacterial community of the critically endangered Panamanian golden frog (*Atelopus zeteki*). Biol Conserv.

[34] Bates KA, Shelton JMG, Mercier VL, Hopkins KP, Harrison XA, Petrovan SO (2019). Captivity and infection by the fungal pathogen batrachochytrium salamandrivorans perturb the amphibian skin microbiome. Front Microbiol.

[35] Eisenhofer R, Helgen KM, Taggart D (2021). Signatures of landscape and captivity in the gut microbiota of Southern Hairy-nosed Wombats (*Lasiorhinus latifrons*). Anim Microbiome.

[36] Wienemann T, Schmitt-Wagner D, Meuser K, Segelbacher G, Schink B, Brune A (2011). The bacterial microbiota in the ceca of Capercaillie (*Tetrao urogallus*) differs between wild and captive birds. Syst Appl Microbiol.

[37] Oliveira BCM, Murray M, Tseng F, Widmer G (2020). The fecal microbiota of wild and captive raptors. Anim Microbiome.

[38] Gibson KM, Nguyen BN, Neumann LM, Miller M, Buss P, Daniels S (2019). Gut microbiome differences between wild and captive black rhinoceros—implications for rhino health. Sci Rep.

[39] Nelson TM, Rogers TL, Carlini AR, Brown MV (2013). Diet and phylogeny shape the gut microbiota of Antarctic seals: a comparison of wild and captive animals. Environ Microbiol.

[40] Tsukayama P, Boolchandani M, Patel S, Pehrsson EC, Gibson MK, Chiou KL (2018). Characterization of wild and captive baboon gut microbiota and their antibiotic resistomes. mSystems.

[41] Xenoulis PG, Gray PL, Brightsmith D, Palculict B, Hoppes S, Steiner JM (2010). Molecular characterization of the cloacal microbiota of wild and captive parrots. Vet Microbiol.

[42] McKenzie VJ, Song SJ, Delsuc F, Prest TL, Oliverio AM, Korpita TM (2017). The effects of captivity on the mammalian gut microbiome. Integr Comp Biol.

[43] Alberdi A, Garazi MB, Aizpurua O. Diversity and compositional changes in the gut microbiota of wild and captive vertebrates: a meta-analysis. Sci Rep. 2021;11:22660. 10.1038/s41598-021-02015-6 PMC8608908 34811423

[44] Clayton JB, Vangay P, Huang H, Ward T, Hillmann BM, Al-Ghalith GA (2016). Captivity humanizes the primate microbiome. Proc Natl Acad Sci USA.

[45] Houtz JL, Sanders JG, Denice A, Moeller AH (2021). Predictable and host-species specific humanization of the gut microbiota in captive primates. Mol Ecol.

[46] Kohl KD, Dearing MD (2014). Wild-caught rodents retain a majority of their natural gut microbiota upon entrance into captivity. Environ Microbiol Rep.

[47] Kohl KD, Brun A, Magallanes M, Brinkerhoff J, Laspiur A, Acosta JC (2017). Gut microbial ecology of lizards: insights into diversity in the wild, effects of captivity, variation across gut regions and transmission. Mol Ecol.

[48] Moeller AH, Suzuki TA, Phifer-Rixey M, Nachman MW (2018). Transmission modes of the mammalian gut microbiota. Science.

[49] Bär J, Leung JM, Hansen C, Loke P, Hall AR, Conour L (2020). Strong effects of lab-to-field environmental transitions on the bacterial intestinal microbiota of Mus musculus are modulated by *Trichuris muris* infection. FEMS Microbiol Ecol.

[50] Chong R, Grueber CE, Fox S, Wise P, Barrs VR, Hogg CJ (2019). Looking like the locals—gut microbiome changes post-release in an endangered species. Anim Microbiome.

[51] van Leeuwen P, Mykytczuk N, Mastromonaco GF, Schulte-Hostedde AI (2020). Effects of captivity, diet, and relocation on the gut bacterial communities of white-footed mice. Ecol Evol.

[52] Moeller AH, Suzuki TA, Lin D, Lacey EA, Wasser SK, Nachman MW (2017). Dispersal limitation promotes the diversification of the mammalian gut microbiota. Proc Natl Acad Sci.

[53] Linnenbrink M, Wang J, Hardouin EA, Künzel S, Metzler D, Baines JF (2013). The role of biogeography in shaping diversity of the intestinal microbiota in house mice. Mol Ecol.

[54] Hu X, Liu G, Li Y, Wei Y, Lin S, Liu S (2018). High-throughput analysis reveals seasonal variation of the gut microbiota composition within forest musk deer (*Moschus berezovskii*). Front Microbiol.

[55] Clayton JB, Al-Ghalith GA, Long HT, Tuan BV, Cabana F, Huang H (2018). Associations between nutrition, gut microbiome, and health in a novel nonhuman primate model. Sci Rep.

[56] Martínez-Mota R, Kohl KD, Orr TJ, Denise DM (2020). Natural diets promote retention of the native gut microbiota in captive rodents. ISME J.

[57] Greene LK, McKenney EA, O’Connell TM, Drea CM (2018). The critical role of dietary foliage in maintaining the gut microbiome and metabolome of folivorous sifakas. Sci Rep.

[58] Narat V, Amato KR, Ranger N, Salmona M, Mercier-Delarue S, Rupp S (2020). A multi-disciplinary comparison of great ape gut microbiota in a central African forest and European zoo. Sci Rep.

[59] Hyde ER, Navas-Molina JA, Song SJ, Kueneman JG, Ackermann G, Cardona C (2016). The oral and skin microbiomes of captive komodo dragons are significantly shared with their habitat. mSystems.

[60] Perofsky AC, Lewis RJ, Abondano LA, Di Fiore A, Meyers LA (2017). Hierarchical social networks shape gut microbial composition in wild Verreaux’s sifaka. Proc Biol Sci.

[61] Tung J, Barreiro LB, Burns MB, Grenier J-C, Lynch J, Grieneisen LE (2015). Social networks predict gut microbiome composition in wild baboons. Elife.

[62] Raulo A, Allen BE, Troitsky T, Husby A, Firth JA, Coulson T (2021). Social networks strongly predict the gut microbiota of wild mice. ISME J.

[63] Caruso R, Ono M, Bunker ME, Núñez G, Inohara N (2019). Dynamic and asymmetric changes of the microbial communities after cohousing in laboratory mice. Cell Rep.

[64] Ridaura VK, Faith JJ, Rey FE, Cheng J, Duncan AE, Kau AL (2013). Gut microbiota from twins discordant for obesity modulate metabolism in mice. Science.

[65] Wernimont SM, Radosevich J, Jackson MI, Ephraim E, Badri DV, MacLeay JM (2020). The effects of nutrition on the gastrointestinal microbiome of cats and dogs: impact on health and disease. Front Microbiol.

[66] Willing BP, Russell SL, Finlay BB (2011). Shifting the balance: antibiotic effects on host-microbiota mutualism. Nat Rev Microbiol.

[67] Eleftheriou A (2021). Implications for one health of anthelmintic use in wildlife conservation programs. EcoHealth.

[68] Leung JM, Loke P (2013). A role for IL-22 in the relationship between intestinal helminths, gut microbiota and mucosal immunity. Int J Parasitol.

[69] Allen-Blevins CR, You X, Hinde K, Sela DA (2017). Handling stress may confound murine gut microbiota studies. PeerJ.

[70] Murakami T, Kamada K, Mizushima K, Higashimura Y, Katada K, Uchiyama K (2017). Changes in intestinal motility and gut microbiota composition in a rat stress model. Digestion.

[71] Reese AT, Dunn RR (2018). Drivers of microbiome biodiversity: a review of general rules, feces, and ignorance. MBio.

[72] Cheng Y, Fox S, Pemberton D, Hogg C, Papenfuss AT, Belov K (2015). The Tasmanian devil microbiome—implications for conservation and management. Microbiome.

[73] Borbón-García A, Reyes A, Vives-Flórez M, Caballero S (2017). Captivity shapes the gut microbiota of andean bears: insights into health surveillance. Front Microbiol.

[74] Sun Y, Sun Y, Shi Z, Liu Z, Zhao C, Lu T (2020). Gut microbiota of wild and captive alpine musk deer (*Moschus chrysogaster*). Front Microbiol.

[75] Yao R, Xu L, Hu T, Chen H, Qi D, Gu X (2019). The “wildness” of the giant panda gut microbiome and its relevance to effective translocation. Glob Ecol Conserv..

[76] Sun C-H, Liu H-Y, Liu B, Yuan B-D, Lu C-H (2019). Analysis of the gut microbiome of wild and captive Père David’s deer. Front Microbiol.

[77] Reese AT, Kearney SM (2019). Incorporating functional trade-offs into studies of the gut microbiota. Curr Opin Microbiol.

[78] Minich D, Madden C, Evans MV, Ballash GA, Barr DJ, Poulsen KP (2021). Alterations in gut microbiota linked to provenance, sex, and chronic wasting disease in white-tailed deer (*Odocoileus virginianus*). BioRxiv.

[79] Rosshart SP, Vassallo BG, Angeletti D, Hutchinson DS, Morgan AP, Takeda K (2017). Wild mouse gut microbiota promotes host fitness and improves disease resistance. Cell.

[80] Blyton MDJ, Soo RM, Whisson D, Marsh KJ, Pascoe J, Le Pla M (2019). Faecal inoculations alter the gastrointestinal microbiome and allow dietary expansion in a wild specialist herbivore, the koala. Anim Microbiome.

[81] Koeppel KN, Bertschinger H, Van Vuuren M, Picard J, Steiner J, Williams D (2006). The use of a probiotic in captive cheetahs (*Acinonyx jubatus*). J S Afr Vet Assoc.

[82] Weimer PJ, Stevenson DM, Mantovani HC, Man SLC (2010). Host specificity of the ruminal bacterial community in the dairy cow following near-total exchange of ruminal contents1. J Dairy Sci.

[83] Hook SE, Northwood KS, Wright A-DG, McBride BW (2009). Long-term Monensin supplementation does not significantly affect the quantity or diversity of methanogens in the rumen of the lactating dairy cow. Appl Environ Microbiol.

[84] Danielsson R, Dicksved J, Sun L, Gonda H, Müller B, Schnürer A (2017). Methane production in dairy cows correlates with rumen methanogenic and bacterial community structure. Front Microbiol.

[85] Clemmons BA, Voy BH, Myer PR (2019). Altering the gut microbiome of cattle: considerations of host-microbiome interactions for persistent microbiome manipulation. Microb Ecol.

[86] Li Y, Hu X, Yang S, Zhou J, Zhang T, Qi L (2017). Comparative analysis of the gut microbiota composition between captive and wild forest musk deer. Front Microbiol.

[87] Gao H, Chi X, Qin W, Wang L, Song P, Cai Z (2019). Comparison of the gut microbiota composition between the wild and captive Tibetan wild ass (*Equus kiang*). J Appl Microbiol.

[88] Ning Y, Qi J, Dobbins MT, Liang X, Wang J, Chen S (2020). Comparative analysis of microbial community structure and function in the gut of wild and captive Amur tiger. Front Microbiol.

[89] Alberdi A, Aizpurua O, Bohmann K, Zepeda-Mendoza ML, Gilbert MTP (2016). Do vertebrate gut metagenomes confer rapid ecological adaptation?. Trends Ecol Evol.

[90] Kohl KD, Weiss RB, Cox J, Dale C, Dearing MD (2014). Gut microbes of mammalian herbivores facilitate intake of plant toxins. Ecol Lett.

[91] Louca S, Polz MF, Mazel F, Albright MBN, Huber JA, O’Connor MI (2018). Function and functional redundancy in microbial systems. Nat Ecol Evol.

[92] Guo W, Ren K, Ning R, Li C, Zhang H, Li D (2020). Fecal microbiota transplantation provides new insight into wildlife conservation. Glob Ecol Conserv..

[93] Martín R, Bermúdez-Humarán LG, Langella P (2016). Gnotobiotic rodents: an in vivo model for the study of microbe–microbe interactions. Front Microbiol.

[94] Iyer N (2016). Methods in microbiome research. Lab Anim.

[95] Greyson-Gaito CJ, Bartley TJ, Cottenie K, Jarvis WMC, Newman AEM, Stothart MR (2020). Into the wild: microbiome transplant studies need broader ecological reality. Proc R Soc B Biol Sci.

[96] Auchtung JM, Robinson CD, Britton RA (2015). Cultivation of stable, reproducible microbial communities from different fecal donors using minibioreactor arrays (MBRAs). Microbiome.

[97] Auchtung JM, Robinson CD, Farrell K, Britton RA (2016). MiniBioReactor Arrays (MBRAs) as a tool for studying *C. difficile* physiology in the presence of a complex community. Methods Mol Biol Clifton NJ.

[98] Bishop J, Hosey G, Plowman A (2013). Handbook of zoo research, guidelines for conducting research in zoos.

[99] Ericsson AC, Hart ML, Kwan J, Lanoue L, Bower LR, Araiza R (2021). Supplier-origin mouse microbiomes significantly influence locomotor and anxiety-related behavior, body morphology, and metabolism. Commun Biol.

[100] Ding R, Goh W-R, Wu R, Yue X, Luo X, Khine WWT (2019). Revisit gut microbiota and its impact on human health and disease. J Food Drug Anal.

[101] Nguyen TLA, Vieira-Silva S, Liston A, Raes J (2015). How informative is the mouse for human gut microbiota research?. Dis Model Mech.

[102] Celi P, Verlhac V, Pérez Calvo E, Schmeisser J, Kluenter A-M (2019). Biomarkers of gastrointestinal functionality in animal nutrition and health. Anim Feed Sci Technol.

[103] Fry TL, Dunbar MR (2007). A review of biomarkers used for wildlife damage and disease management.

[104] Olifiers N, Jansen AM, Herrera HM, Bianchi RC, D’Andrea PS, Mourão GM (2015). Co-infection and wild animal health: effects of trypanosomatids and gastrointestinal parasites on coatis of the Brazilian pantanal. PLoS ONE.

[105] Knutie SA (2020). Food supplementation affects gut microbiota and immunological resistance to parasites in a wild bird species. J Appl Ecol.

[106] Aouissi HA, Ababsa M, Gaagai A, Bouslama Z, Farhi Y, Chenchouni H (2021). Does melanin-based plumage coloration reflect health status of free-living birds in urban environments?. Avian Res.

[107] Lane EP, Miller S, Lobetti R, Caldwell P, Bertschinger HJ, Burroughs R (2012). Effect of diet on the incidence of and mortality owing to gastritis and renal disease in captive cheetahs (*Acinonyx jubatus*) in South Africa. Zoo Biol.

[108] Rosshart SP, Herz J, Vassallo BG, Hunter A, Wall MK, Badger JH (2019). Laboratory mice born to wild mice have natural microbiota and model human immune responses. Science.

[109] De Araujo Lima Constantino P (2016). Deforestation and hunting effects on wildlife across Amazonian indigenous lands. Ecol Soc.

[110] Taylor-Brown A, Booth R, Gillett A, Mealy E, Ogbourne SM, Polkinghorne A (2019). The impact of human activities on Australian wildlife. PLoS ONE.

[111] Daszak P, Cunningham AA, Hyatt AD (2000). Emerging infectious diseases of wildlife—threats to biodiversity and human health. Science.

[112] Thornes T (2016). Animals and climate change. J Anim Ethics..

[113] Alfano N, Courtiol A, Vielgrader H, Timms P, Roca AL, Greenwood AD (2015). Variation in koala microbiomes within and between individuals: effect of body region and captivity status. Sci Rep.

[114] Allan N, Knotts TA, Pesapane R, Ramsey JJ, Castle S, Clifford D (2018). Conservation implications of shifting gut microbiomes in captive-reared endangered voles intended for reintroduction into the wild. Microorganisms.

[115] Benno Y, Itoh K, Miyao Y, Mitsuoka T (1987). Comparison of fecal microflora between wild Japanese monkeys in a snowy area and laboratory-reared Japanese monkeys. Jpn J Vet Sci.

[116] Bik EM, Costello EK, Switzer AD, Callahan BJ, Holmes SP, Wells RS (2016). Marine mammals harbor unique microbiotas shaped by and yet distinct from the sea. Nat Commun.

[117] De Jesús-Laboy KM, Godoy-Vitorino F, Piceno YM, Tom LM, Pantoja-Feliciano IG, Rivera-Rivera MJ (2012). Comparison of the fecal microbiota in feral and domestic goats. Genes.

[118] Delport TC, Power ML, Harcourt RG, Webster KN, Tetu SG (2016). Colony location and captivity influence the gut microbial community composition of the Australian sea lion (*Neophoca cinerea*). Appl Environ Microbiol.

[119] Delsuc F, Metcalf JL, Parfrey LW, Song SJ, González A, Knight R (2014). Convergence of gut microbiomes in myrmecophagous mammals. Mol Ecol.

[120] Eigeland K, Lanyon J, Trott D, Ouwerkerk D, Blanshard W, Milinovich G (2012). Bacterial community structure in the hindgut of wild and captive dugongs (*Dugong dugon*). Aquat Mamm.

[121] Frankel JS, Mallott EK, Hopper LM, Ross SR, Amato KR (2019). The effect of captivity on the primate gut microbiome varies with host dietary niche. Am J Primatol.

[122] Guan Y, Zhang H, Gao X, Shang S, Wu X, Chen J (2016). Comparison of the bacterial communities in feces from wild versus housed sables (*Martes zibellina*) by high-throughput sequence analysis of the bacterial 16S rRNA gene. AMB Express.

[123] Guan Y, Yang H, Han S, Feng L, Wang T, Ge J (2017). Comparison of the gut microbiota composition between wild and captive sika deer (*Cervus nippon hortulorum*) from feces by high-throughput sequencing. AMB Express.

[124] Guo W, Mishra S, Wang C, Zhang H, Ning R, Kong F (2019). Comparative study of gut microbiota in wild and captive giant pandas (*Ailuropoda melanoleuca*). Genes.

[125] Hale VL, Tan CL, Niu K, Yang Y, Zhang Q, Knight R (2019). Gut microbiota in wild and captive Guizhou snub-nosed monkeys, *Rhinopithecus brelichi*. Am J Primatol.

[126] Haworth SE, White KS, Côté SD, Shafer ABA (2019). Space, time and captivity: quantifying the factors influencing the fecal microbiome of an alpine ungulate. FEMS Microbiol Ecol.

[127] Kong F, Zhao J, Han S, Zeng B, Yang J, Si X (2014). Characterization of the gut microbiota in the Red Panda (*Ailurus fulgens*). PLoS ONE.

[128] Milovic A, Bassam K, Shao H, Chatzistamou I, Tufts DM, Diuk-Wasser M (2020). Lactobacilli and other gastrointestinal microbiota of *Peromyscus leucopus*, reservoir host for agents of Lyme disease and other zoonoses in North America. PLoS ONE.

[129] Moustafa MAM, Chel HM, Thu MJ, Bawm S, Htun LL, Win MM (2021). Anthropogenic interferences lead to gut microbiome dysbiosis in Asian elephants and may alter adaptation processes to surrounding environments. Sci Rep.

[130] Nakamura N, Amato KR, Garber P, Estrada A, Mackie RI, Gaskins HR (2011). Analysis of the hydrogenotrophic microbiota of wild and captive black howler monkeys (*Alouatta pigra*) in palenque national park, Mexico. Am J Primatol.

[131] Prabhu VR, Wasimuddin, Kamalakkannan R, Arjun MS, Nagarajan M (2020). Consequences of domestication on gut microbiome: a comparative study between wild gaur and domestic Mithun. Front Microbiol.

[132] Schwab C, Cristescu B, Northrup JM, Stenhouse GB, Gänzle M (2011). Diet and environment shape fecal bacterial microbiota composition and enteric pathogen load of grizzly bears. PLoS ONE.

[133] Tang J, Wang C, Zhang H, Zhao J, Guo W, Mishra S (2020). Gut microbiota in reintroduction of giant panda. Ecol Evol.

[134] Uenishi G, Fujita S, Ohashi G, Kato A, Yamauchi S, Matsuzawa T (2007). Molecular analyses of the intestinal microbiota of chimpanzees in the wild and in captivity. Am J Primatol.

[135] Wasimuddin, Menke S, Melzheimer J, Thalwitzer S, Heinrich S, Wachter B (2017). Gut microbiomes of free-ranging and captive Namibian cheetahs: diversity, putative functions and occurrence of potential pathogens. Mol Ecol.

[136] Xiao Y, Xiao G, Liu H, Zhao X, Sun C, Tan X (2019). Captivity causes taxonomic and functional convergence of gut microbial communities in bats. PeerJ.

[137] San Juan PA, Castro I, Dhami MK (2021). Captivity reduces diversity and shifts composition of the Brown Kiwi microbiome. Anim Microbiome.

[138] Scupham AJ, Patton TG, Bent E, Bayles DO (2008). Comparison of the cecal microbiota of domestic and wild turkeys. Microb Ecol.

[139] Xie Y, Xia P, Wang H, Yu H, Giesy JP, Zhang Y (2016). Effects of captivity and artificial breeding on microbiota in feces of the red-crowned crane (*Grus japonensis*). Sci Rep.

[140] Campos P, Guivernau M, Prenafeta-Boldú FX, Cardona L (2018). Fast acquisition of a polysaccharide fermenting gut microbiome by juvenile green turtles *Chelonia mydas* after settlement in coastal habitats. Microbiome.

[141] García-De la Peña C, Garduño-Niño E, Vaca-Paniagua F, Díaz-Velásquez C, Barrows CW, Gomez-Gil B (2019). Comparison of the fecal bacterial microbiota composition between wild and captive bolson tortoises (*Gopherus flavomarginatus*). Herpetol Conserv Biol.

[142] Sandri C, Correa F, Spiezio C, Trevisi P, Luise D, Modesto M (2020). Fecal microbiota characterization of seychelles giant tortoises (*Aldabrachelys gigantea*) living in both wild and controlled environments. Front Microbiol.

[143] Tang G-S, Liang X-X, Yang M-Y, Wang T-T, Chen J-P, Du W-G (2020). Captivity influences gut microbiota in crocodile lizards (*Shinisaurus crocodilurus*). Front Microbiol.

[144] Tong Q, Liu X-N, Hu Z-F, Ding J-F, Bie J, Wang H-B (2019). Effects of captivity and season on the gut microbiota of the brown frog (*Rana dybowskii*). Front Microbiol.

[145] Edenborough KM, Mu A, Mühldorfer K, Lechner J, Lander A, Bokelmann M (2020). Microbiomes in the insectivorous bat species Mops condylurus rapidly converge in captivity. PLoS ONE.

[146] Schmidt E, Mykytczuk N, Schulte-Hostedde A (2019). Effects of the captive and wild environment on diversity of the gut microbiome of deer mice (*Peromyscus maniculatus*). ISME J.

[147] Dhanasiri AKS, Brunvold L, Brinchmann MF, Korsnes K, Bergh Ø, Kiron V (2011). Changes in the intestinal microbiota of wild Atlantic cod *Gadus morhua* L. Upon Captive Rearing Microb Ecol.

